# SAILOR: perceptual anchoring for robotic cognitive architectures

**DOI:** 10.1038/s41598-024-84071-2

**Published:** 2025-01-02

**Authors:** Miguel Á. González-Santamarta, Francisco J. Rodrıguez-Lera, Vicente Matellan-Olivera, Virginia Riego del Castillo, Lidia Sánchez-González

**Affiliations:** Robotics Group, Department of Mechanic Engineering, Computer and Aerospace Sciences, University of León, 24006 León, Spain

**Keywords:** Computational science, Computer science, Information technology, Software

## Abstract

Symbolic anchoring is an important topic in robotics, as it enables robots to obtain symbolic knowledge from the perceptual information acquired through their sensors and maintain the link between that knowledge and the sensory data. In cognitive-based robots, this process of transforming sub-symbolic data generated by sensors to obtain and maintain symbolic knowledge is still an open problem. To address this issue, this paper presents SAILOR, a framework for symbolic anchoring integrated into ROS 2. SAILOR aims to maintain the link between symbolic data and perceptual data in real robots over time. It provides a semantic world modeling approach using two deep learning-based sub-symbolic robotic skills: object recognition and matching function. The object recognition skill allows the robot to recognize and identify objects in its environment, while the matching function enables the robot to decide if new perceptual data corresponds to existing symbolic data. This paper describes the proposed method and the development of the framework, as well as its integration in MERLIN2 (a hybrid cognitive architecture fully functional in robots running ROS 2) and the validation of SAILOR using public datasets and a real-world scenario.

## Introduction

The use of cognitive architectures ^1–3^ as a mechanism for generating robot behaviors is broadly accepted. There are three types of cognitive architectures: symbolic, based on the principles of symbolic AI that include knowledge representation, reasoning, and planning modules; emergent, based on the principles of emergence and complex systems where behavior emerges from interactions between components; and hybrid, a combination of both. Although there is no well-defined degree of hybridization between symbolic and emergent concepts, usually they work with symbolic information, using for instance the PDDL (Planning Domain Description Language) ^4^.

In the robotics community, the Robot Operating System (ROS)^5^ is widely regarded as the de facto standard framework, while PDDL is widely accepted for representing the symbolic knowledge in robotic systems. There are several PDDL-based tools used in practical cognitive architectures, however, two of them are well known: ROSPlan ^6^ and PlanSys2 ^7^. Both apply PDDL to represent the real world of the robot by creating the objects and the attributes of the world. Afterward, it is used by symbolic planners such as POPF (Partial Order Planning Forwards) ^8^ to generate plans to achieve the goals of the robots.

The issue up here is how the model of the world serves the architecture and how it is updated over time. As a result, the research question faced in this paper is how to perform the task of creating and maintaining the correspondences between symbolic data and sensor data in a cognitive architecture for robots.

Generating PDDL knowledge from raw sensory data is not straightforward. Thus, perception is the process of converting raw sensory data into cognitive architectures’ internal representation, particularly symbolic knowledge. Whereas symbolic anchoring ^9^ is the process of creating and maintaining the correspondence between symbols and sensor data that refer to the same physical objects. Knowledge creation is needed not only, but also, knowledge maintenance. This is an aspect of the Symbolic Knowledge Grounding ^10^ that is the problem of how to ground the meanings of symbols used by the robot. The process of grounding symbols to real-world objects by a physical agent interacting in the real is known as Physical Symbol Grounding ^11^. Thus, the flow of how to model PDDL knowledge from information provided by sensors to store in the world model database is defined.

Therefore, this paper presents SAILOR (Symbolic AnchorIng from perceptual data for rOs2-based Robots) a component that creates and maintains real-time knowledge in the ROS 2 ecosystem, which acts as the middleware that facilitates communication between the sensory data and the symbolic anchoring system. The authors have selected ROS 2 over ROS 1 due to the programmed End-Of-Life of ROS 1 scheduled in 2025. Thus, SAILOR is integrated into a ROS 2-based hybrid cognitive architecture providing the capability to do symbolic anchoring. SAILOR combines two sub-symbolic skills: one to obtain information by recognizing the objects that the robot sees and the other one to decide if there is new knowledge or if old knowledge has to be updated. Besides, As a result, knowledge about the real world is obtained and maintained in real-time as the robot interacts with the world rather than only using innate knowledge manually created before running the cognitive architectures.

### Contributions and article overview

The main contribution of this paper is the development of a framework for performing symbolic anchoring in ROS 2. Specifically, this research provides the following contributions:

1. An enhanced symbolic anchoring pipeline: An approach based on state-of-the-art techniques is proposed, utilizing object detection followed by physical feature extraction from point clouds. This differs from existing methods in the literature, which typically employ point cloud segmentation followed by image classification. The proposed method improves object detection while reducing the computational complexity associated with processing the entire point cloud.
2. A novel matching function: A matching function that incorporates object tracking and deep learning is introduced for symbolic anchoring. This function determines whether an object is being encountered by the robot for the first time, offering improved accuracy in object recognition.
3. Integration of the SAILOR solution: The proposed symbolic anchoring solution has been implemented within a cognitive architecture in ROS 2, demonstrating its applicability in robotic systems.
4. Comprehensive evaluation: The matching function for symbolic anchoring is evaluated using state-of-the-art indoor and outdoor datasets, as well as through real-world experiments involving a physical robot in actual environments.

## Related works

Symbolic anchoring is the process of creating and maintaining the link between symbolic data and sensor data. Symbolic anchoring systems are based on extracting features from physical objects. Then, they are used to check if new perceived objects correspond with known objects. This mechanism is commonly known as the matching function. As a result, the symbolic anchoring problem ^9^ is related to how to perform symbolic anchoring in an artificial intelligence system. In fact, symbolic anchoring is a special case of Physical Symbol Grounding ^11^ where symbolic data is maintained and updated in time. The symbolic anchoring problem involves different areas, such as psychology, cognition, linguistics and computer science. This makes symbolic anchoring a complex process.

There are initial works in symbolic anchoring that use fuzzy logic to implement the matching function and the grounding, such as^12^. In later works, we can find one of the basic symbolic anchoring pipelines presented in^13^. It shows a basic system for symbolic anchoring using only visual features to implement the matching function used to check if new objects have to be stored in the knowledge base. This system was composed of a perceptual layer, where sensory data is generated and processed to extract features; an anchoring layer, where the grounding and symbolic anchoring processes take place; and a knowledge representation layer, which contains a knowledge base. This approach uses SIFT (Scale-invariant feature transform) ^14^ to produce visual features to check if new objects are obtained. The grounding is based on describing the physical objects using color, object class, semantic localization and spatial relations.

The work ^15^ presents a method for modeling the semantic environment of a robot using probabilistic multiple hypothesis anchoring (PMHA), which includes the matching function. The method uses probabilistic reasoning to update the robot’s understanding of the environment as it receives new sensory information, however, there is not a clear symbolic anchoring architecture of the complete system. The features used as input for its algorithm are color images and object shapes. It also presents a grounding skill that describes the objects using their size and their color.

Another alternative is presented in^16^. This work proposed a method for improving the symbolic anchoring of objects in the real world through the use of learned actions. The feature used in the matching function is the Euclidean distance between two objects and the classification coefficient. Both features are used in the matching function that is based on a formula with a threshold for each input. Besides, the presented framework is based on integrating actions and perception to improve the accuracy of symbolic anchoring in situations where visual perception alone is unreliable. The framework is provided with machine learning models that learn actions that can be used to disambiguate objects thus improving the symbolic anchoring of objects in the real world. Another case of using object poses is presented in^17^. In this work, the features used are again the Euclidean distance and classification coefficient. These two features are fed into the matching function that is based on a Support Vector Machine (SVM) plus the use of the Hungarian Method ^18^ to assign each perception to an existing anchor. The SVM is trained with a dataset created by the authors using a mobile robot.

More complex matching functions can be found in symbolic anchoring systems. In cite ^19^, authors present a matching function based on machine learning techniques. The data used to train the model is composed of five similarities: object class, color histogram, distance, size and timestamp. The dataset was collected indoors using a fixed camera that was in front of the used robot manipulator. Then, several models are trained, such as SVM and KNN.

According to the literature, the use of symbolic anchoring in robots is still a problem to be faced. There are different approaches but all of them have in common the feature extraction from sensory data and the implementation of a matching function that allows knowing if new perceptions correspond with known objects. In this work, we present a new symbolic anchoring from perceptual data, based on ROS 2, that uses deep learning to carry out the matching function.

In computer vision, there is a wide variety of architectures available for object detection^20^. It is approached from three points of view: the neural networks backbone such as AlexNet ^21^ or ResNet ^22^, detectors based on two-stage anchors such as Faster R-CNN ^23^ or one-stage anchors such as YOLO ^24^, and detectors based on transforms such as DETR ^25^. However, YOLOv8 ^26^ has become a leading reference, since the software developed by Ultralytics allows fast, accurate and easy detection, classification, and segmentation.

Moreover, some tasks in the computer vision field have similarities with symbolic anchoring from the perceptual data. These tasks are object tracking ^27^ and image retrieval ^28^. Symbolic anchoring systems can be improved by using object tracking, as it locates and follows a particular object over a sequence of frames. Symbolic anchoring also requires extracting high-level symbolic representations from sensory data to decide whether something exists in current knowledge. In this sense, it is similar to image retrieval in that it searches for images in a database that are similar to a given image by extracting certain features and applying machine learning algorithms to find a match. However, symbolic anchoring from perception involves the extraction of high-level symbolic representations from sensory data.

One popular technique used for computing image similarity is the Siamese Convolutional Neural Networks ^29^. These networks learn a similarity metric by training on pairs of images. This approach has shown promising results in various image retrieval applications^30–33^, allowing for effective comparison and matching of images based on their visual content.

The approach proposed in this paper creates a symbolic understanding of the observed environment by defining meaningful symbols of objects and their relationships. It goes beyond visual recognition as it offers a more abstract and interpretable representation for robotic reasoning and decision-making. While image retrieval is focused on matching and categorization, symbolic anchoring goes further, extracting symbolic meaning and updating the existing knowledge, which enables robots to reason about their surroundings in a more semantically rich manner.

## SAILOR proposal

The Materials and Methods section of this paper aims to detail the components and processes used to develop a symbolic anchoring from perception system for robots. This system leverages the principles of cognitive architecture to allow robots to anchor their perceptions to symbolic representations, enabling them to process and understand the environment in a more human-like manner. The section is divided into two main subsections: Formalization of Symbolic Anchoring from Perceptual Data and SAILOR pipeline. These subsections will provide a comprehensive understanding of the methodology and procedures used to build the symbolic anchoring system, as well as the datasets used.

**Figure 1:**
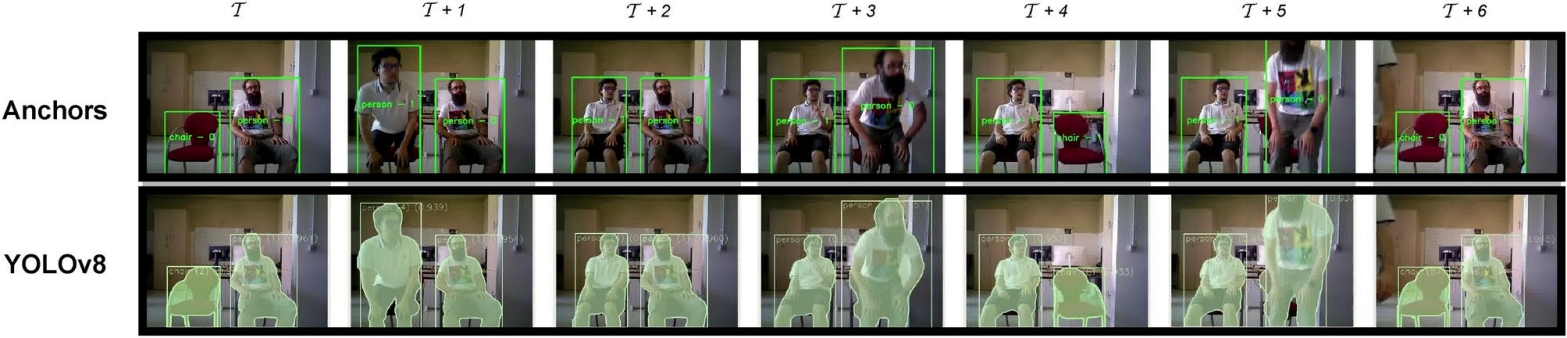
Example of SAILOR during time. The first row represents the current anchors of SAILORS while the second row represents the detection obtained from YOLOv8. Each anchor is given a symbolic name, which are person-0, person-1, chair-0 and chair-1.

An example of applying SAILOR is presented in Fig. 1. The first row represents the current anchors of SAILOR, each one with a symbolic name like person-0, while the second row represents the detection obtained from YOLOv8. This example starts with two anchors, a person, person-0, and a chair, chair-0. Then, new anchors appear when a new person, person-1, and a new chair, chair-1, are detected. Finally, when the previous anchors disappear and reappear, they are maintained using the same symbolic names, which are person-0 and chair-0.

### Formalization of symbolic anchoring from perceptual data

Symbolic anchoring is the task of creating and maintaining in time the correspondences between symbolic data and sensor data. This correspondence is also called percepts, which are data structures used to define physical objects. Following^9,13,34,35^, the symbolic anchoring is composed of three systems:

- Perceptual system: This system is in charge of generating percepts from the data obtained from the real world. It includes a set of percepts, $\Pi = \{\pi _1, \pi _2,...\}$. Each percept has a set of measurable features, $\phi _i$ with values in the domain $D(\phi _i)$. As a result, the perceptual system includes a set of features, $\Phi = \{\phi _1, \phi _2,...\}$, used to describe each percept.
- Anchoring system: This is the system in charge of updating the symbolic knowledge using percepts. This correspondence is represented by the data structure called anchor, $\alpha _i$. Thus, this system has a set of anchors, $A = \{\alpha _1, \alpha _2,...\}$.
- Symbolic system: This system is in charge of maintaining symbolic knowledge and using it to reason about the actions needed to achieve certain goals. Symbolic knowledge is stored in a knowledge base composed of four sets:

- Set of classes $C = \{c_1, c_2,...\}$ that describes the classes of objects that can appear in the problem.
- Set of objects $O = \{o_1, o_2,...\}$ that contains the objects of the problem.
- Set of predicates $P = \{p_1, p_2,...\}$ that contains the attributes of the world.
- Set of facts $F = \{f_1, f_2,...\}$ that described the the world.

Symbolic anchoring ^9^ also has a *predicate grounding relation* $G \subseteq P \times \phi \times D(\phi )$. This relation is in charge of encoding features $\phi _i$ from each $\pi _i$ using the properly predicates $p_i$ to create the facts $f_i$. On the one hand, percepts can be described using visual features, such as color histograms, descriptors and semantic object categories. On the other hand,^17^ uses physical features, such as 3D position, 3D size and orientation. Finally, a combination of both, visual and physical features, can be used, as in the case of^19^.

#### The functionalities of symbolic anchoring

In the symbolic anchoring process, anchors $\alpha _i$ can be created both top-down and bottom-up. Bottom-up approaches are based on events from the perceptual system (e.g. new percepts obtained $\pi _i$ from object recognition) whose data can be linked to existing anchors. Nevertheless, top-down takes place when symbolic data needs to be related to a percept.

The maintenance of anchors takes place at each cycle of SAILOR when new percepts are created. The new percepts are compared with the existing anchors and two different situations can occur. Those percepts that match an anchor are used to update the anchor and the symbolic data with the new information. On the contrary, if there is no match, new anchors are created. To check if a percept matches an anchor, a matching function *M* (Eq.  1) is used. This function takes as input a percept $\pi _i$ and an anchor $\alpha _i$ and returns the degree of matching.

1 $$\begin{aligned} M: \pi \times \alpha \rightarrow [0, 1] \end{aligned}$$

There are four main functionalities associated with symbolic anchoring as presented in the literature (^19,36^):

- Acquire: This process initiates new anchors whenever new percepts are received and do not match any existing anchor. It takes each new percept $\pi _i$ and each existing anchor $\alpha _i$ and computes the matching degree using the matching function *M*. For each percept that does not match an anchor, symbolic data (objects and facts) is created using the *predicate grounding relation* *G*.
- Find: This process takes an object $o_i$ and the facts $f_i$ that describe that object and returns an anchor $\alpha _i$. Then, that anchor is compared against the existing anchors and current percepts. If there is an anchor that matches, that anchor is selected. A new anchor is created if there is no match.
- Re-acquire: This process is intended to extend the definition of a matching anchor $\alpha _i$ from the timestamp $\tau$ to timestamp $\tau + \kappa$. It is based on taking the matching percepts and updating the anchors and the symbolic data over time.
- Track: This process is based on taking an anchor $\alpha _i$ defined in timestamp $\tau$ and extends its definition to timestamp $\tau + \kappa$. This can be performed by using the re-acquire functionality if a match takes place or by predicting the future state of the anchor after some elapsed time from the last observation.

### Pipeline

**Figure 2:**
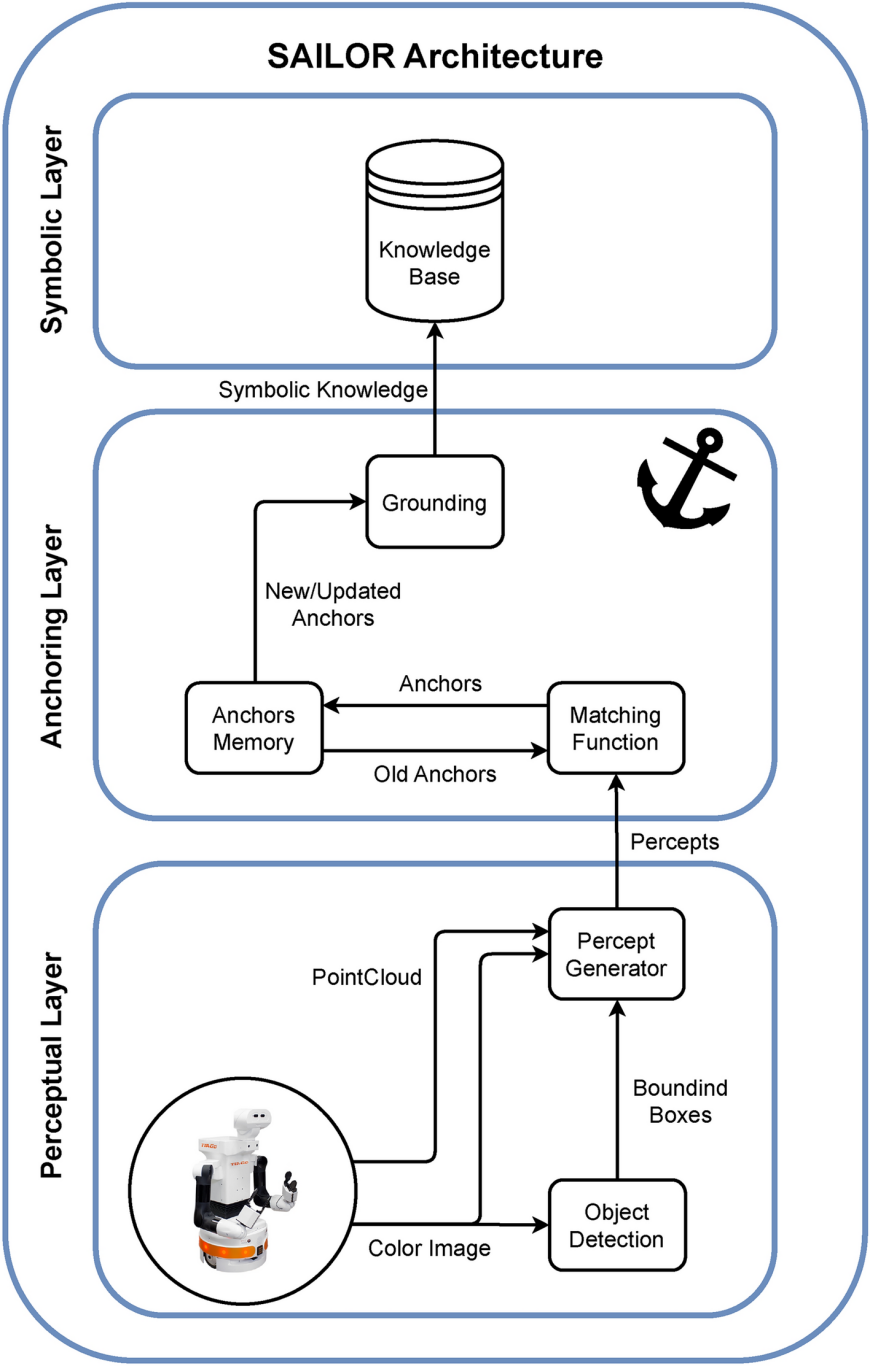
SAILOR’s framework. It is composed of a symbolic layer, an anchoring layer, and a perceptual layers.

SAILOR (Symbolic AnchorIng from perceptuaL data for rOs2-based Robots) is the symbolic anchoring system integrated into the cognitive architecture. SAILOR’s framework, which is a bottom-up symbolic anchoring framework, is presented in Fig. 2. It is divided into three layers: perceptual, anchoring and symbolic layers as described in^13^.

#### Perceptual layer

This layer generates the percepts using the sensory data. To do this, an RGB-D camera is used. The color images captured from the camera are processed by an object detection system, which is a sub-symbolic skill of the robot. Then, a set of features of each percept is extracted from the color images, the obtained bounding boxes of the objects and the point clouds captured from the camera. Considering the features employed by the existing solutions analyzed in the related works section, we have selected the following five visual and physical features to describe each percept:

- Class of the object: this feature is obtained from the object detection skill. In this work, YOLOv8 (YOLOv8m) ^26^ was used to process color images obtained from the robot to detect objects in the environment since it was the best object detection system when this work took place.
- Tracking ID: this feature is obtained by applying the tracking algorithm ByteTrack ^37^ to the output of YOLOv8. The default configuration of ByteTrack is employed.
- Cropped image: this feature is the image obtained after cropping the original frame from the camera using the bounding box detected by YOLOv8.
- Position of the object: this feature is the object centroid in a 3D space of the detected object in meters. From the obtained bounding box, the pixel location of its centroid (cx,cy) is determined. Next, these pixels are converted to the 3D camera coordinate system by using the obtained point cloud. Then, this position is transformed from the local coordinate system of the camera to the global coordinate system of the robot.
- Size of the object: this feature is the size of the object, in meters, represented as a box. The measurements are obtained by calculating the maximum and minimum, for each 3D axis, in the point cloud data within the limits of the bounding box. The data of the point cloud, the points, has to be aligned with the data of the image, the pixels.
- Timestamp: this feature is the timestamp of the obtained image.

#### Anchoring layer

This is the layer in charge of managing the anchors, this means anchor robot perceptions to symbolic representations. Thus, it applies the matching function to each percept received from the perceptual layer. If these percepts, which belong to a certain frame, are new (they do not match an existing anchor), new anchors are created. Those percepts that match an anchor provide updated information to that anchor and its corresponding symbolic knowledge. This implies applying the acquire and reacquire functionalities respectively.

The symbolic anchoring procedure is presented in Algorithm 1. In the initial case, when there are no anchors, all received percepts are acquired, which involves anchor creations. In the following cases, the new percepts are used to create a matching table which is a matrix that is composed of the probabilities of each percept (rows) to match each anchor (columns).

**Algorithm 1:**
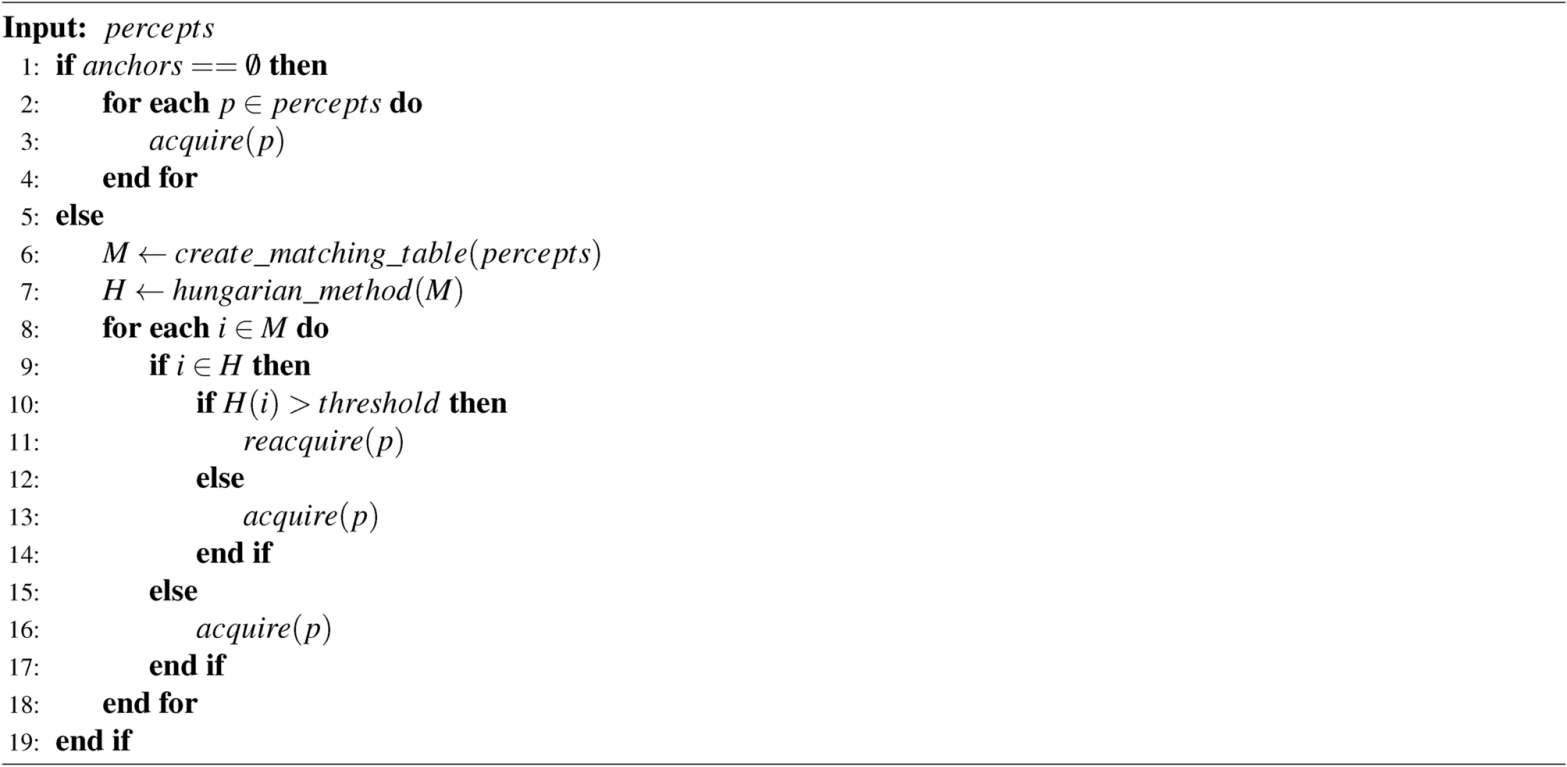
SAILOR’s symbolic anchoring algorithm.

The procedure to create the matching table is shown in Algorithm 2. It is based on applying the matching function to each candidate–that is, each new percept–and to the existing anchors. Each pair of percept-anchor is compared using their features. As a result, the table obtained is a matrix $N\times M$, where *N* is the number of new percepts and *M* is the number of existing anchors. Each cell contains the matching value for each pair of percept-anchor which is a value in the range [0, 1].

Then, the Hungarian method ^18^ is applied to the matching table. This algorithm is used to solve the assignment problem, finding the optimal assignment of agents to tasks in a cost-minimization or profit-maximization scenario. The output is the rows and columns that correspond to the associations. As a result, each percept is associated with its corresponding anchor.

Finally, the pairs of percept-anchor of the associations with a matching value greater than a threshold, that is 0.5, are the reacquire cases. However, the pairs with a value below the threshold are acquiring cases. There are also acquiring cases when new percepts are not part of the result of the Hungarian Method. This can happen if the number of new percepts is greater than the number of existing anchors.

**Algorithm 2:**
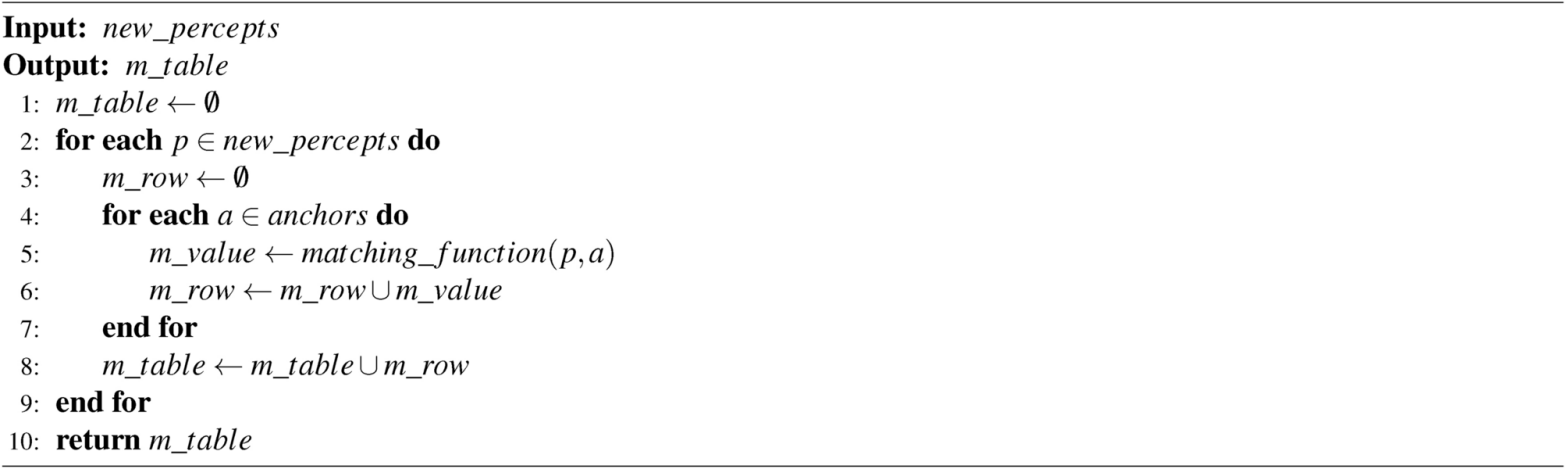
Create matching table algorithm.

#### Matching function

The matching function implemented in the presented solution checks if new perceived objects correspond with known objects (stored percepts). Thus, a match is obtained if each pair of percept-anchor tracking IDs is the same as the new one.

The comparison of each pair of percept-anchor gives the input features of the neural network which are the following:

- Same object class: this feature is a boolean that indicates if the classes of the percept and the anchor are the same.
- Cropped images: these RGB images correspond to the cropped images of the percept and the anchor. They are used to measure visual similarity. The images are preprocessed following the next steps:

- The images are cropped to a size of 224 $\times$ 224 pixels. This means that a square region of the image is selected, discarding the remaining parts.
- Then, they are resized to a size of 256 $\times$ 256 applying bilinear interpolation, which is a technique to estimate pixel values in the resized image based on the surrounding pixels. Bilinear interpolation helps in preserving the visual quality of the images during the resizing process. This step ensures that all the input images have the same size for consistency.
- The pixel values of the images are normalized using mean and standard deviation values of ImageNet, which are [0.485, 0.456, 0.406] and [0.229, 0.224, 0.225] respectively. This normalization centers the pixel values around zero, which helps in reducing the effect of lighting variations in the images; and scales the pixel values to have a standard deviation of 1, which aids in reducing the impact of color channel variations.

- Distance: this feature is the $L^2$-distance in meters between the positions of the percept ($\pi$) and the anchor ($\alpha$). Similar to the work^19^, the distance is normalized using 2.
  2 $$\begin{aligned} distance = e^{-L^2(\pi ^{pos}, \alpha ^{pos})} \end{aligned}$$
- Scale factor: this feature is the scale factor between the sizes of the percept ($\pi$) and the anchor ($\alpha$). It is computed using the Jaccard Similarity whose formula is presented in 3.
  3 $$\begin{aligned} scale\_factor = \frac{\sum _{1}^{3}min(\pi ^{size}, \alpha ^{size})}{\sum _{1}^{3}max(\pi ^{size}, \alpha ^{size})} \end{aligned}$$
- Time: this feature is the time, in seconds, between the timestamps of the percept ($\pi$) and the anchor ($\alpha$). This value is also normalized following the work^19^ by using 4.
  4 $$\begin{aligned} time = \frac{2}{1 + e^{abs(\pi ^{time} - \alpha ^{time})}} \end{aligned}$$

**Figure 3:**
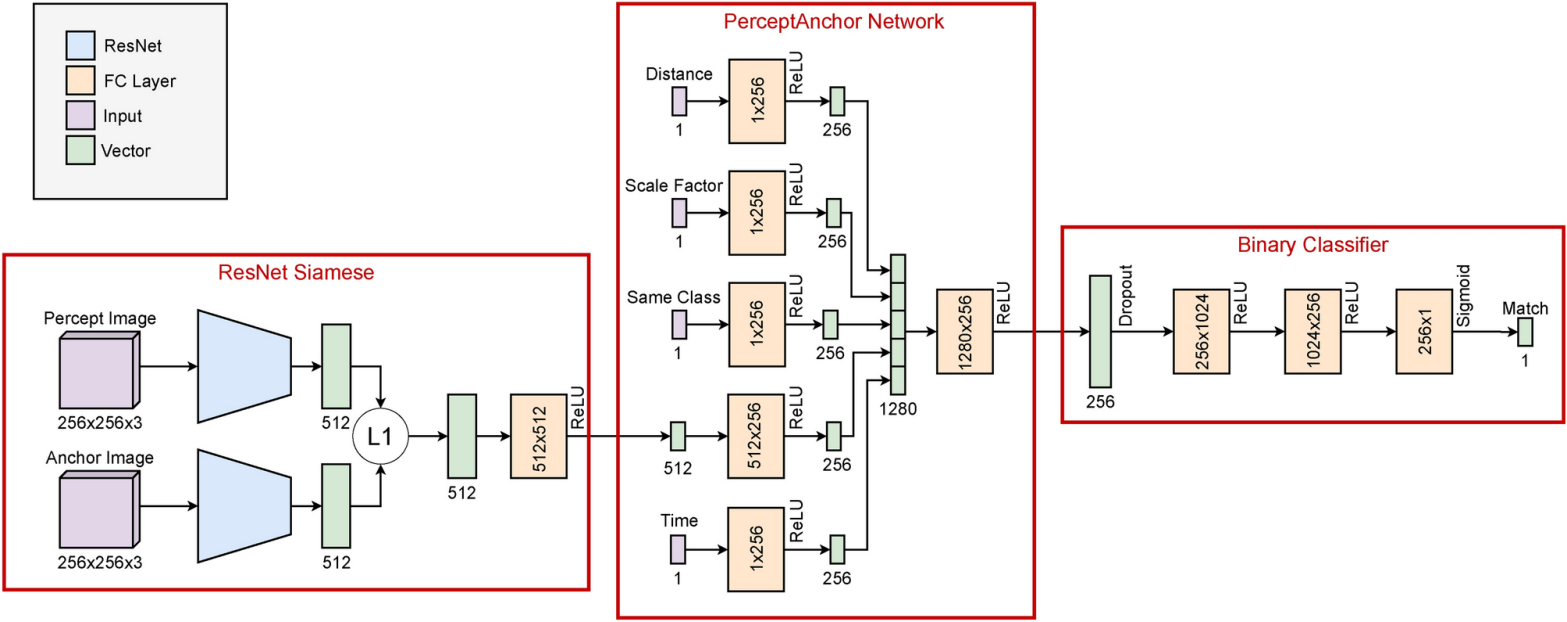
Neural network used to implement the matching function of SAILOR. It is divided into three components: ResNet Siamese, which produces the similarity between two images as a feature vector; PerceptAnchor Network, which encodes each pair of percept-anchor; and Binary Classifier, which classifies each encoded pair as reacquired or acquired.

The neural network used in this work is presented in Fig. 3. This network is composed of three modules:

- ResNet Siamese: following the Siamese Convolutional Neural Network used to measure the similarity of two images, we have used two frozen ResNet-18 ^38^ networks to extract features of the cropped images of each pair of percept-anchor. Then, the $L^1$-distance is applied to the outputs of the ResNet-18. This result is fed into a fully connected layer. As a result, the comparison of the two images from the pair percept-anchor is obtained.
- PerceptAnchor Network: this network is in charge of encoding and concatenating the five features that describe each pair percept-anchor. There is a fully connected layer for each input feature (class, ResNet Siamese output, distance, size, timestamps). Then, the outputs are concatenated and fed into another fully connected layer.
- Binary Classifier: this network is a Multi-Layer Perceptron (MLP) responsible for classifying the pair percept-anchor as a reacquire. It uses the output of the previous network as its input and returns a value between 0 and 1, thanks to the sigmoid function, that indicates the matching degree between the percept and the anchor.

In the process of training a neural network, choosing the appropriate optimizer and learning rate is crucial to achieving optimal performance. In this study, the training of this neural network was carried out using an Adam optimizer and a learning rate of 0.00001. Adam ^39^ is a popular optimizer that combines the benefits of two other optimization techniques, namely, Adagrad and RMSprop. This optimizer has been shown to work well in a wide range of deep-learning tasks and is known for its efficient convergence rate. Meanwhile, the learning rate determines the step size taken in each iteration of the optimization process. A low learning rate may cause convergence to be slow, while a high learning rate can lead to overshooting the optimal solution.

#### Symbolic layer

This layer consists of the knowledge base responsible for storing the symbolic knowledge that the robot uses to understand and interact with its environment. The symbolic knowledge is represented using the PDDL, a formalism that allows the robot to reason about its world in a structured manner. This layer plays a crucial role in maintaining and updating the knowledge necessary for the robot’s planning and decision-making processes, enabling it to perform tasks in a dynamic environment.

We have adopted the knowledge base from KANT ^40^, which provides flexibility in how the symbolic knowledge is managed. Specifically, the knowledge base can be implemented either as a ROS 2 node or as a MongoDB database. In the former case, the knowledge is stored in memory, allowing for quick access but limited persistence. In the latter, MongoDB offers a more durable solution, ensuring that the symbolic anchoring system retains knowledge even after system restarts or interruptions, thus providing long-term consistency in the robot’s understanding of the world.

The symbolic knowledge within KANT consists of types, objects, predicates, and propositions-concepts that closely mirror the structure of PDDL. Each real-world object is linked to a symbolic object, and its characteristics are expressed through predicates and propositions. This structured representation enables the robot to abstract and interpret the physical world in symbolic terms, allowing it to execute complex plans by reasoning over these symbolic entities. As a result, the robot can not only identify objects but also understand their relationships and roles within various tasks, enhancing its cognitive abilities in real-world scenarios. This symbolic representation facilitates the integration of perception and reasoning, providing a bridge between low-level sensor data and high-level decision-making.

## Experimental setup

This section introduces the experimentation carried out in this work. Thus, the experimentation is based on comparing SAILOR in different datasets and in a real-world environment. In addition, the proposed matching function formed by the previously explained neural network is also compared with other solutions that can behave as a matching function computing if an object has been already acquired or is a new one. So we have considered the four machine learning classifiers presented in work^19^, which are Gaussian Naive Bayes, K-Nearest Neighbors (KNN), Multi-Layer Perceptron (MLP), and Support Vector Machines (SVM). These four classifiers are trained using the same features as the SAILOR neural network except for the image tensors which are replaced by the color correlation between image histograms as in^19^.

Although ROS 2 and MERLIN2 provide critical support for real-time robotic integration, the scientific contributions of this paper focus on the theory and algorithm of SAILOR. ROS 2 serves as a middleware, facilitating communication between components and enabling real-time data processing. It is mainly used in this work for integrating SAILOR into the TIAGo robot, allowing access to perceptual data and object detection functions in dynamic environments. However, this paper does not propose novel developments in ROS 2 itself. Similarly, MERLIN2 acts as a cognitive architecture within which SAILOR operates, but its main function here is to manage high-level robotic tasks. Therefore, we will retain our focus on SAILOR’s contributions to perceptual anchoring, particularly its pipeline and symbolic anchoring methods, which include object detection and matching using deep learning.

The experimental setup for this study encompassed several main aspects: SAILOR and ROS 2, MERLIN2, Datasets, real-world setup and hardware setup. Firstly, the ROS 2 components that compose SAILOR and its integration in MERLIN2 are presented. Secondly, diverse datasets comprising real-world data were employed to evaluate the performance of the proposed approach. Thirdly, the setup for the real-world setup is presented. Lastly, a carefully configured hardware setup was employed.

**Figure 4:**
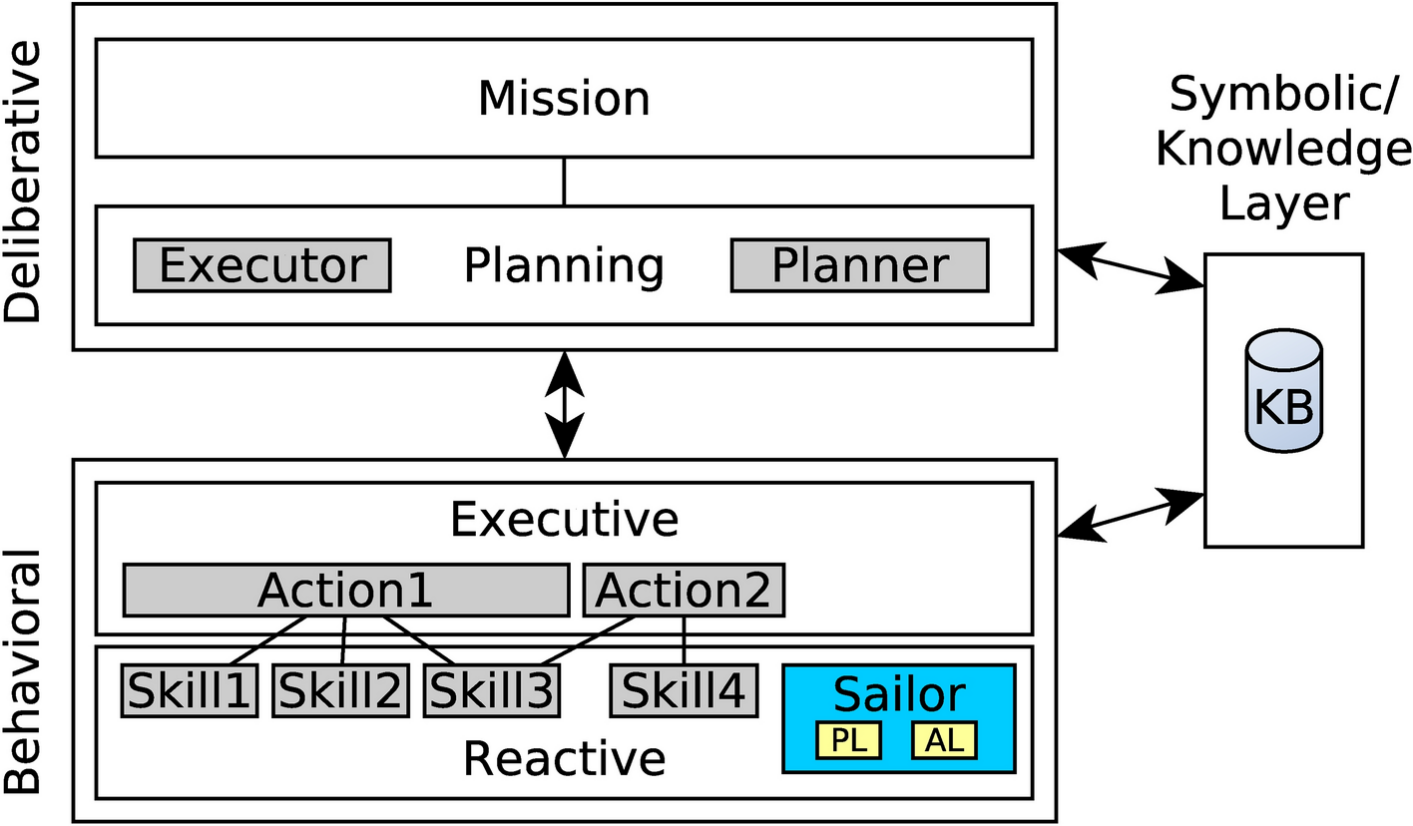
MERLIN2 architecture showing SAILOR as a robot skill.

### Cognitive architecture

Cognitive architectures employed in robotics need a way to dynamically obtain knowledge from the environment, which is facing the symbolic anchoring problem. An example of this is presented in^41^. It proposes a method for generating symbolic representations of the world from sensory data inside a cognitive architecture. The symbolic representations are stored in the Knowledge Base, nested in a Symbolic Layer, using PDDL ^4^. Then, that knowledge is used to produce plans that solve the goals of the robot.

In this research, MERLIN2 architecture ^42^ is used. The architecture is composed of two main systems, Deliberative and Behavioural, that are divided into two layers each. The Deliberative is composed of the Mission Layer and the Planning Layer, where the Knowledge Base can be found. The Behavioural is composed of the Executive Layer and the Reactive Layer, where robot skills can be found.

Figure 4 illustrates how SAILOR components are integrated into the MERLIN2 architecture. The sub-symbolic skills, Perceptual Layer (PL) and Anchoring Layer (AL), of SAILOR are integrated into the Reactive Layer from the Behavioral as any other skill available in the robot such as NAV2 and Text-to-Speech. The Symbolic Layer of SAILOR corresponds with the Knowledge Base of the Planning Layer.

Therefore, MERLIN2 is only used to store the symbolic knowledge created by SAILOR and it is not included in the evaluation of this work since we want to focused on the symbolic knowledge creation.

### SAILOR in ROS 2

**Figure 5:**
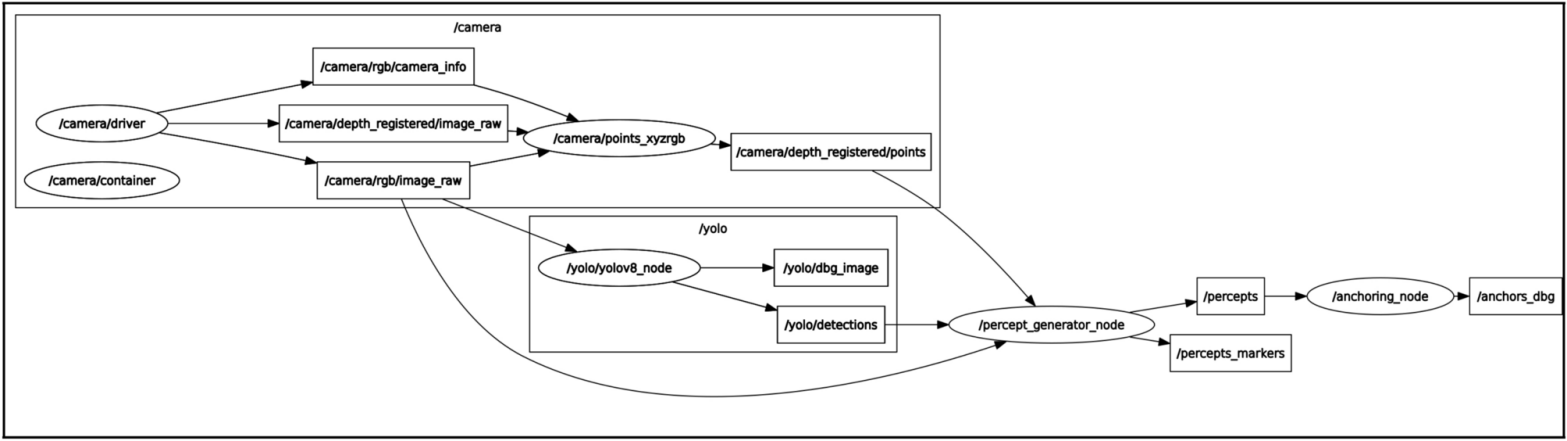
Rosgraph of SAILOR, which includes YOLOv8 and camera nodes.

SAILOR pipeline has been implemented in ROS 2 for its integration into a real robot called TIAGo. Figure 5 shows the rosgraph of SAILOR. It is composed of camera nodes to produce RGB images and point cloud data, the YOLOv8 node, the percept generator node and the anchoring node. The percept generator node is subscribed to RGB images, point cloud data and YOLOv8 detections to produce percepts. Then, the anchoring node is subscribed to these percepts and applies the symbolic anchoring procedures.

### Datasets

The matching function presented in this work is based on a deep learning solution so it is mandatory to collect a dataset in order to train the neural network. In previous machine learning-based works, custom datasets have been created. For instance, in^19^ a new dataset is created using a custom labeling tool. This dataset describes each pair of percept-anchor using five similarities (classification, histogram, distance, size, time). However, all data is obtained in scenarios where the robot, a robotic arm, is fixed to a table. Besides, in^17^ a custom dataset is created using a mobile robot. In this case, pairs of percept-anchor are described using the classification values and distances.

**Algorithm 3:**
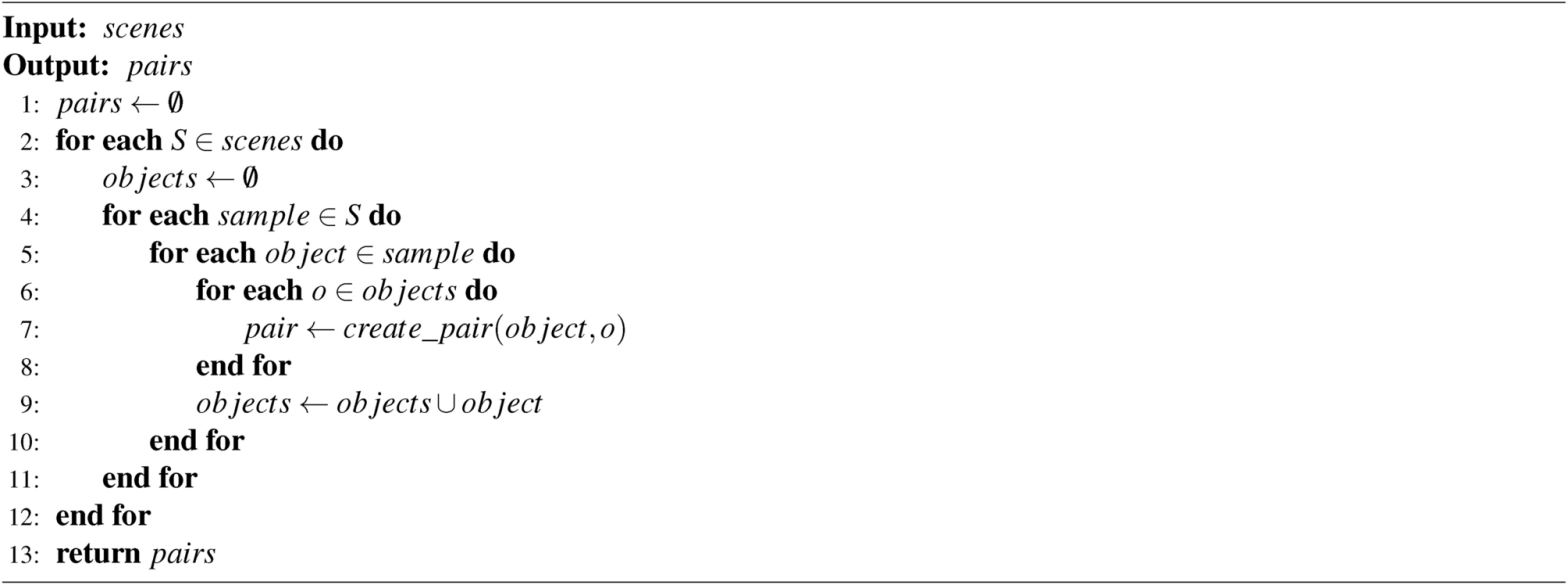
Create dataset algorithm.

Several existing and public datasets can be used. For instance, KITTI dataset ^43^, is a dataset intended to be used in several tasks such as autonomous driving and object detection. It comprises traffic scenarios recorded with diverse sensor modalities. Another similar dataset is nuScenes ^44^ which is a large public dataset for autonomous driving. It contains scenes of images, LIDAR data and ground truth that can be used in several tasks. It includes a Python library to access the data and apply transforms to the positions and sizes of detected objects.

One recent indoor dataset that can be used is MOTFront ^45^. It provides photo-realistic RGB-D images with their corresponding instance segmentation masks, class labels, 3D bounding boxes and 3D poses. The scenes were captured in indoor scenarios with furniture.

With all of this, we have created three new datasets from nuScenes and MOTFront datasets. We have chosen these two datasets to achieve a more diverse solution thanks to the indoor and outdoor data. This would allow us to evaluate the scalability and generality of our learned matching function across different scenarios.

To create the new datasets, the procedure presented in Algorithm 3 is applied to each scene of the datasets. This way, each object of each sample from the scenes is used to create the pairs of percept-anchor. These pairs are created by calculating the five input features of the neural network presented previously.

The previous algorithm is applied to the full MOTFront dataset. In the case of nuScenes, scenes 1, 2, 4, 5, 6, 7, 8, 41, 42 and 43 have been used for training; scenes 3, 12, 13, 14 and 15 for validation; and scenes 1069, 1070, 1071, 1072 and 1073 for testing. Then, both resulting datasets were merged to create the third dataset it is called *Mix*. The resulting datasets are characterized in Table 1.

**Table 1:** Datasets created using nuScenes and MOTFront data with the number of samples (pairs of percept-anchor) in the training, validation and test splits.

Dataset	Train	Val	Test
nuScenes	429615	33172	52806
MOTFront	469928	104274	116655
Mix	899543	137446	169461

### Leon@Home testbed

**Figure 6:**
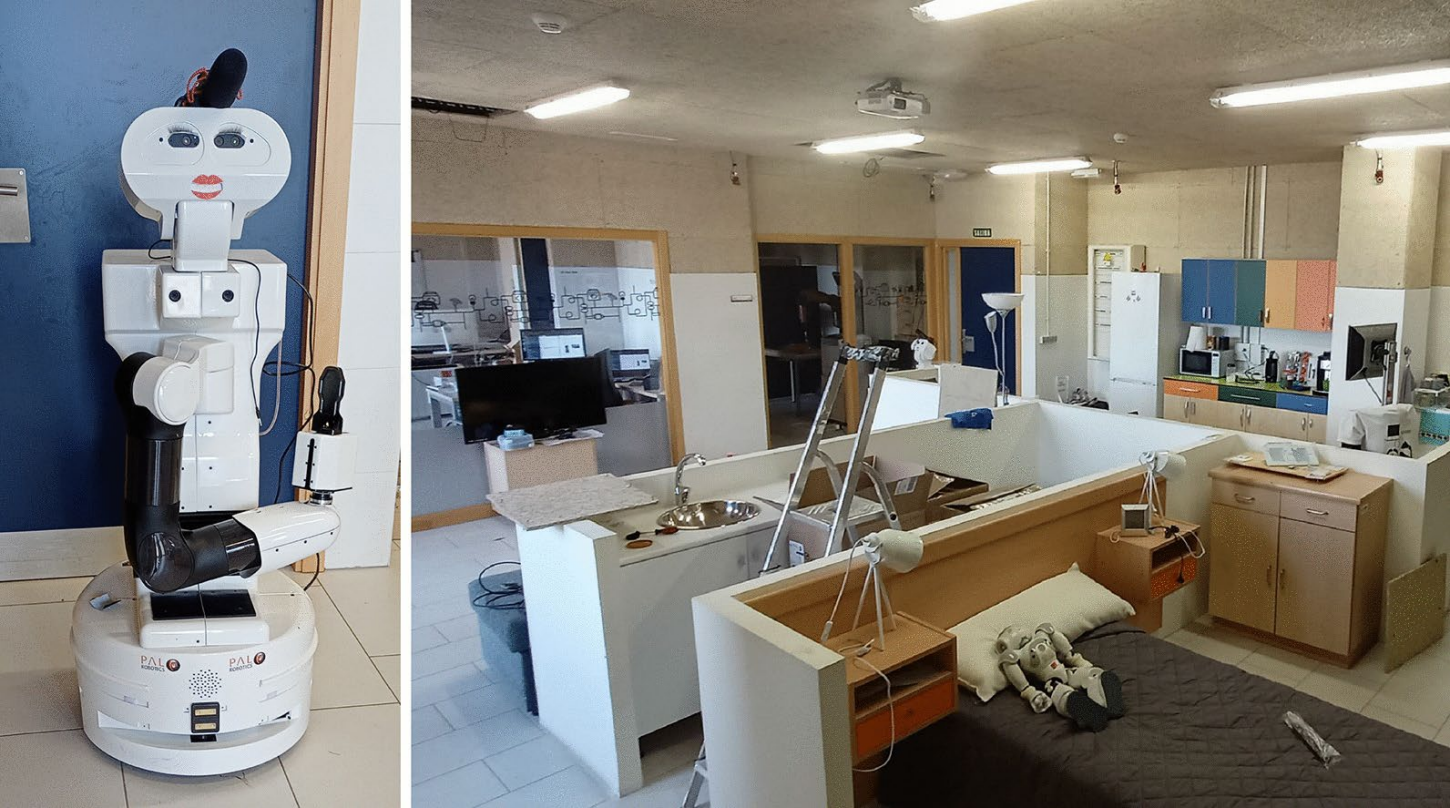
Real-world robot and environment used in experimentation. The robot used is TIAGo, a service robot with a differential mobile base, equipped with a LiDAR, and a torso, equipped with an RGB-D camera, speakers and microphone. The real-world environment is the apartment Leon@Home Testbed of the Robotics Group of the University of León.

Besides the experiments with the datasets to compare with the existing solutions, we have designed an experiment in a real environment. This experiment is based on using the TIAGo robot, presented in Fig. 6, in a real-world environment while running the SAILOR. Specifically, the real-world environment is the mock-up apartment Leon@Home Testbed also illustrated in Fig. 6. This is a certified testbed of the European Robotics League (ERL) located in the Robotics Group of the University of Leon. It is used to test mobile service robots in a realistic environment. The apartment is composed of a living room, kitchen, bedroom, and bathroom. We have recorded ROS 2 rosbags while the robot is navigating through the apartment while people walk and interact with the environment. These rosbags, which contain the robot data, are used to recreate the robot data employing SAILOR to obtain the pairs of percept-anchor that are going to be used with the matching function. Thus, 63,474 pairs of percept-anchor are obtained to evaluate SAILOR and compare it with the existing machine learning classifiers.

### Hardware setup

All anchored data were acquired with the use of a TIAGo mobile Robot which is equipped with an Asus Xtion Pro live RGB-D sensor. TIAGo Robot runs ROS Melodic and bridges for interfacing with ROS 2 humble of an external laptop with an Intel(R) i7-8750H CPU, 8 GB RAM and a GTX 1060 Nvidia. Moreover, the training and test were performed using a remote machine with an AMD EPYC 7302P CPU, 256 GB RAM and a Quadro RTX 8000 Nvidia.

## Evaluation

This evaluation section discusses the results of the trained models. Specifically, the SAILOR neural network is trained on three previous datasets to test its ability to anchor symbols to sensory data. The authors examined the performance of the same algorithm, the Matching Function, under the three datasets. The evaluation of the quality of the trained models was carried out by such criteria as a confusion matrix.

Afterward, these values are then used to calculate different evaluation metrics, such as accuracy, precision, recall and F1-score. Accuracy measures the proportion of correct predictions, while precision measures the proportion of true positives among all positive predictions. Recall measures the proportion of true positives among all actual positives, while the F1-score is the harmonic mean of precision and recall.

### Results

Table 2 presents average symbolic anchoring classification accuracy, precision, recall and F1-score for the SAILOR models based on the proposed neural network when using the two existing datasets–nuScenes and MOTFront–and the *Mix* dataset formed by the combination of both. It also gathers the data obtained from the Leon@Home scenario.

**Table 2:** Resulting average classification accuracy together with precision, recall and F1-score for each SAILOR models tested in our approach to performing the symbolic anchoring functionalities in nuScene, MOTFront and *Mix* datasets and in the Leon@Home scenario.

	Accuracy	Precision	Recall	F1-score
nuScenes dataset				
nuScenes	0.9869	0.8358	0.7624	0.7974
MOTFront	0.9667	0.9064	0.9967	0.9494
Mix	0.9730	0.9037	0.9858	0.9430
Leon@Home	0.9946	0.9775	0.9695	0.9735
MOTFront dataset				
nuScenes	0.9655	0.4933	0.7211	0.5858
MOTFront	0.9754	0.9562	0.9656	0.9609
Mix	0.9723	0.9256	0.9542	0.9397
Leon@Home	**0.9958**	**0.9930**	**0.9658**	**0.9792**
Mix dataset				
nuScenes	0.9864	0.8854	0.6870	0.7737
MOTFront	0.9857	0.9688	0.9862	0.9774
Mix	0.9859	0.9658	0.9722	0.9690
Leon@Home	0.9949	0.9905	0.9590	0.9745

Significant values are in bold.

The accuracy metric defines the total number of correctly classified data over the total number of data. However, this metric is not enough for non-balanced datasets. Precision defines how accurate is the model out of those predicted positives and measures how many of them are actually positives. This metric is a good measure to determine when the cost of a False Positive is high, this means measuring how many elements are we detecting wrongly. Recall defines how many of the Actual Positives our model captures by labeling it as Positive (True Positive). This metric helps us to select the best model when there is a high cost associated with False Negative. In this case, when we do not acquire an anchor that is present in the scene. F1-score is a metric to be applied if it is needed to seek a balance between Precision and Recall. In these large datasets would be an uneven class distribution (a considerable number of certain Negatives).

Comparing the results of SAILOR models, we got that the best results in our real-world scenario are achieved with the model trained with the MOTFront dataset. Besides, the nuScene tests are the ones that got the worst results. Moreover, the best results in the nuScene test are obtained using the nuScene dataset, which also obtains high results in the MOTFront test. The model trained with the MOTFront dataset obtains the worst results in the nuScene test but high accuracy, precision, recall and F1-score in its own test.

On the other hand, Tables 3, 4, 5, 6 present average classification accuracy, precision, recall and F1-score for Bayes, KNN, SVM and MLP models when using nuScenes, MOTFront and *Mix* datasets and the data obtained from the Leon@Home scenario.

**Table 3:** Resulting average classification accuracy together with precision, recall and F1-score for each Bayes model tested in our approach to performing the symbolic anchoring functionalities in nuScene, MOTFront and Mix datasets and in the Leon@Home scenario.

	Accuracy	Precision	Recall	F1-score
nuScenes dataset				
nuScenes	0.9999	0.9994	0.9994	0.9994
MOTFront	0.8105	0.8809	0.4568	0.6017
Mix	0.8695	0.8911	0.4821	0.6257
Leon@Home	0.9143	**0.9971**	0.1628	0.2799
MOTFront dataset				
nuScenes	0.9770	0.9965	0.3220	0.4867
MOTFront	0.9904	0.9716	0.9986	0.9849
Mix	0.9862	0.9720	0.9671	0.9695
Leon@Home	**0.9930**	0.9957	**0.9363**	**0.9652**
Mix dataset				
nuScenes	0.9884	0.9933	0.6629	0.7952
MOTFront	0.9915	0.9750	0.9983	0.9865
Mix	0.9859	0.9658	0.9722	0.9690
Leon@Home	0.9905	0.9755	0.9827	0.9791

Significant values are in bold.

**Table 4:** Resulting average classification accuracy together with precision, recall and F1-score for each KNN model tested in our approach to performing the symbolic anchoring functionalities in nuScene, MOTFront and Mix datasets and in the Leon@Home scenario.

	Accuracy	Precision	Recall	F1-score
nuScenes dataset				
nuScenes	0.9934	0.9118	0.8899	0.9007
MOTFront	0.9887	0.9689	0.9959	0.9822
Mix	0.9901	0.9663	0.9909	0.9785
Leon@Home	**0.9935**	0.9763	**0.9599**	**0.9681**
MOTFront dataset				
nuScenes	0.9886	1.000	0.66406	0.7981
MOTFront	0.9983	0.9978	0.9967	0.9973
Mix	0.9953	0.9979	0.9812	0.9895
Leon@Home	0.9918	0.9893	0.9302	0.9588
Mix dataset				
nuScenes	0.9934	0.9196	0.8820	0.9004
MOTFront	0.9982	0.9977	0.9965	0.9971
Mix	0.9967	0.9942	0.9912	0.9926
Leon@Home	0.9909	**0.9764**	0.9342	0.9548

Significant values are in bold.

**Table 5:** Resulting average classification accuracy together with precision, recall and F1-score for each MLP model tested in our approach to performing the symbolic anchoring functionalities in nuScene, MOTFront and *Mix* datasets and in the Leon@Home scenario.

	Accuracy	Precision	Recall	F1-score
nuScenes dataset				
nuScenes	0.9918	1.0000	0.7574	0.8619
MOTFront	0.8697	0.9987	0.5849	0.7377
Mix	0.9077	0.9988	0.5930	0.7441
Leon@Home	0.9627	0.9923	0.6400	0.7781
MOTFront dataset				
nuScenes	0.9873	1.0000	0.6260	0.7700
MOTFront	0.9982	0.9976	0.9966	0.9971
Mix	0.9948	0.9976	0.9793	0.9884
Leon@Home	0.9914	**0.9953**	0.9200	0.9562
Mix dataset				
nuScenes	0.9882	0.8776	0.7574	0.8131
MOTFront	0.9966	0.9916	0.9977	0.9946
Mix	0.9940	0.9870	0.9865	0.9867
Leon@Home	**0.9927**	0.9832	**0.9453**	**0.9639**

Significant values are in bold.

**Table 6:** Resulting average classification accuracy together with precision, recall and F1-score for each SVM model tested in our approach to performing the symbolic anchoring functionalities in nuScene, MOTFront and *Mix* datasets and in the Leon@Home scenario.

	Accuracy	Precision	Recall	F1-score
nuScenes dataset				
nuScenes	0.9999	0.9994	0.9966	0.9980
MOTFront	0.8157	0.9034	0.4612	0.6107
Mix	0.8731	0.9118	0.4862	0.6342
Leon@Home	0.9280	0.9984	0.2962	0.4569
MOTFront dataset				
nuScenes	0.9788	0.9985	0.3740	0.5441
MOTFront	0.9981	0.9980	0.9961	0.9970
Mix	0.9921	0.9980	0.9670	0.9823
Leon@Home	**0.9893**	**0.9978**	0.8970	**0.9447**
Mix dataset				
nuScenes	0.9862	0.8551	0.7127	0.7774
MOTFront	0.9978	0.9964	0.9966	0.9965
Mix	0.9942	0.9909	0.9834	0.9871
Leon@Home	0.9792	0.8621	**0.9482**	0.9032

Significant values are in bold.

Comparing the results of the Bayes, KNN, MLP and SVM models we got that the datasets affect in different ways the models. For instance, the Bayes model gets the best results in the Leon@Home scenario by using the MOTFront dataset while the KNN model gets the best results by using the nuScene dataset. In the case of the MLP model, the best precision for the Leon@Home is obtained by using the MOTFront dataset and the best accuracy, recall and F1-score are obtained by using the Mix dataset. The SVM achieves the best accuracy, precision and F1-score when using the MOTFront dataset but the best recall is obtained by using the Mix dataset.

Additionally, comparing the SAILOR, Bayes, KNN, MLP and SVM models, we got that the MOTFront dataset is the one that influences most in the training since the models trained with that dataset obtain the best results in our real-world scenario, which is the Leon@Home Testbed. Additionally, the SAILOR model trained with MOTFront presents the best results in the Leon@Home scenario, except for the precision that is achieved by the SVM model.

Finally, the SAILOR’s deep learning model offers significant advantages over KNN for the matching function in robotic environments. Unlike KNN, which relies on simple distance metrics and struggles with high-dimensional, complex data, SAILOR’s neural network can model intricate relationships between object features, such as visual appearance, size, and spatial positioning. This allows it to handle dynamic scenarios like changes in lighting, orientation, and partial occlusion, which are common in real-world robotic applications. Moreover, SAILOR’s learned feature representations make it more robust to noisy or incomplete data and offer better scalability for real-time processing in large datasets, where KNN’s performance degrades due to its reliance on distance calculations for every query. Thus, SAILOR’s model obtains better results, that is a higher F1-score, than the KNN model in the real-world scenario Leon@Home.

### Contribution evaluation

This research proposed four main contributions that have been validated:

1. It presented an updated symbolic anchoring pipeline based on state-of-the-art works. The presented pipeline is based on object detection followed by percept generation, which implies using the bounding boxes to get physical features from the point cloud of the camera.
2. The matching function based on deep learning for an anchoring system achieves more than 96% accuracy in all cases tested.
3. The evaluation of datasets and models for symbolic anchoring is being validated by mixing nuScenes (outdoors) and MOTFront (indoors) datasets. The resulting mixed dataset is available at Hugging Face (https://huggingface.co/datasets/unileon-robotics/sailor).
4. A set of ROS 2 components have been validated and tested in a real robotic Platform TIAGo in the Leon@Home Testbed. These components are publicly available in a GitHub Repository (https://github.com/MERLIN2-ARCH/sailor).
5. The proposed matching function based on deep learning has been compared with the machine learning models presented in^19^ (Bayes, KNN, MLP and SVM) obtaining that the SAILOR model gets better results in the Leon@Home scenario, which is the Leon@Home scenario.

## Conclusions

In this work, we have successfully developed SAILOR, a comprehensive suite of software components for ROS 2, designed to facilitate symbolic anchoring within the cognitive architecture of robotic systems. Our approach introduces a novel symbolic anchoring pipeline that first performs object detection, followed by point cloud-based physical feature extraction, a reversal of the conventional process found in existing state-of-the-art methods. This restructured pipeline offers a more efficient and accurate framework for symbolic anchoring.

Central to our approach is the development of a matching function based on a neural network architecture. This function integrates a ResNet Siamese network, and a PerceptAnchor Network and concludes with a Binary Classifier. By leveraging state-of-the-art datasets and testing within the Leon@Home Testbed, we were able to evaluate its performance. Comparative analyses with other machine learning models, including Gaussian Naive Bayes, K-Nearest Neighbors (KNN), Multi-Layer Perceptron (MLP), and Support Vector Machines (SVM), demonstrate that our proposed matching function outperforms these alternatives in the Leon@Home scenario. Particularly, we have found that the MOTFront dataset provides the most robust results. Our experimental findings further affirm that our approach is well-suited for real-world applications, specifically in the Leon@Home scenario, where it consistently achieved superior performance.

Looking ahead, future work will focus on the development of more advanced datasets that incorporate increasingly realistic and complex scenarios, enhancing the robustness of the anchoring process. Additionally, we plan to refine anchor management strategies within SAILOR, for instance in terms of optimizing the conditions under which anchors should be dynamically removed. Besides, we aim to conduct extensive testing in real-world environments, such as the Leon@Home Testbed, to further validate and enhance the robot’s behavior through improved symbolic anchoring. These efforts will contribute to the continued evolution of cognitive robotic systems, making them more adaptable and effective in diverse operational settings. Finally, future research will explore the resulting impact of generating behaviors using MERLIN2 and SAILOR. This will allow us to better understand how robotic behaviors can be enhanced through the symbolic anchoring capabilities of SAILOR in conjunction with the high-level reasoning provided by MERLIN2.

## Data Availability

The data is available on Hugging Face (https://huggingface.co/datasets/unileon-robotics/sailor).

## Code availability

The data is available on GitHub (https://github.com/MERLIN2-ARCH/sailor).
